# The Role of Grit in Students’ L2 Engagement in the English as a Foreign Language Classroom

**DOI:** 10.3389/fpsyg.2021.749844

**Published:** 2021-09-07

**Authors:** Juan Liu

**Affiliations:** Department of Foreign Languages, Inner Mongolia University of Finance and Economics, Hohhot, China

**Keywords:** foreign language class, grit, students L2 engagement, policymakers and academic experts, scholastic contexts

## Abstract

Due to the rapid development of teaching and learning English as a Foreign Language (EFL), on the one hand, and the arrival of positive psychology (PP) in the process of language education, on the other hand, student engagement has been burgeoned and got a noteworthy role in the academic field. The present review attempts to investigate the relationship of grit with students’ L2 engagement, by examining both backgrounds and consequences of grit. Consequently, the effectiveness of findings for policymakers and academic experts is discussed, along with the prominence of strengthening grit in the scholastic contexts in order to cultivate character in learners and improve their prospects.

## Introduction

With the enhancement of positive psychology (PP), scholars have regularly discerned its significant role in the second language acquisition (SLA) field (MacIntyre and Mercer, 2014; MacIntyre et al., 2019a). In the psychology and learning domain, PP has represented abundant attention in recent years (Seligman et al., 2006; Karlen et al., 2019; Wang et al., 2021). Seligman and Csikszentmihalyi (2014) acknowledged the objectives of PP and delineated the three aspects in the PP study, namely constructive individual behaviors, progressive involvements, and positive associations. Gabryś-Barker and Gałajda (2016) highlighted that those students’ behaviors, encouraging feelings, and learning situations are three significant facets that affect students’ presentation in language education.

Positive psychologists have tried to improve the attentiveness of how regular persons flourish under more non-threatening circumstances to catalyze a shift in the emphasis of consciousness from concentration only on replacing the wickedest issues in life to shape constructive eminences (Seligman and Csikszentmihalyi, 2000). One of the desired learners’ involvements in the educational performance field is their engagement, which is hypothesized as covering the emotional, cognitive, and behavioral scopes (Mercer, 2019). Behavioral engagement talks about the students’ actual disposition to take part in tasks, while emotional engagement is perceived as learners’ emotional state of commitment and connection to a task (Mercer, 2019). In addition, cognitive engagement occurs when one is effectively and emotionally faced, and captivated by an individual’s work (Lei et al., 2018). However, regardless of its short history, engagement has extended remarkable prevalence in educational investigation entrenched in PP (Mercer and Dörnyei, 2020).

Mastering a second/foreign language (L2) is a broad practice; therefore, L2 students will during this process, without a doubt, face disappointments, and demoralizations. Nonetheless, L2 students’ reactions to these disappointments can be unique. Some may see disappointment as a sign of an absence of insight and fitness and probably will not place more conviction into learning a language (Khajavy et al., 2021). Others may see disappointment as a natural part of language learning and focus more on learning L2; accordingly, L2 students’ observations of their L2 learning capacity are identified with their strength in L2 training.

As declared by Khajavy et al. (2021), the connection among these impressions of L2 learning capacity and exertion in L2 education could be clarified by grit, as a positive, non-intellectual attribute, which has as of late gotten a lot of consideration.

As stated by Keegan (2017), grit has been recommended to assume a critical part in assisting learners with putting forth the ideal attempt to further develop their English language capability despite the difficulties they face. The idea of grit as an indicator and basic component of success and accomplishment has been of significant interest in character and learning psychology throughout previous years. Grit has additionally been receiving attention in further diverse areas, containing business, medical services, and instruction (Sudina et al., 2021). Grit is viewed as an individual quality regular in leaders, and a significant forerunner of progress and greatness in each domain paying little mind to skill or ability (Duckworth and Yeager, 2015).

Additionally, grit is a self-guideline and non-intellectual behavioral characteristic made out of two center aspects: (a) persistence of effort and (b) constancy of interests (Duckworth and Quinn, 2009). The former is the effortful quest for objectives despite mishaps. The latter is responsibility and energy devoted to one objective. A mix of these two characteristics has been anticipated for several years, as grit is planned to catch the long-conversed collaboration between the “energy and persistence” needed to influence enduring objectives (Duckworth, 2016).

Since the effective authority of an L2 is profoundly subject to L2 students’ supported exertion and energy throughout an extensive time (Dörnyei and Ushioda, 2013), research of grit and its connection to learners’ language accomplishment turns out to be promptly pertinent in SLA. By comprising commitment and accomplishment as results, the discrepancy impacts between grit and accomplishment and grit and commitment can be inspected; thereby, revealing different advantages that grit might bring to learners’ education. As learner attentiveness has been demonstrated to be a noteworthy school element for learners’ accomplishment and well-being (Wang and Eccles, 2012), commitment will be analyzed as an autonomous scholarly result of grit.

Studies have proved that grit is significantly associated with desire, resilience, well-being, enthusiasm, pleasure, and life fulfillment (Calo et al., 2019; Karlen et al., 2019; Moen and Olsen, 2020; Shakir et al., 2020), while it is negatively interrelated to burnout, hopelessness, and stress (Datu et al., 2019; Moen and Olsen, 2020; Shakir et al., 2020).

For years, grit, as a non-cognitive personality attribute, had a fundamental role in student accomplishment (Duckworth and Quinn, 2009) and spiritual paradigms such as despair and enthusiasm (Steinmayr et al., 2018; Datu et al., 2019). Keegan (2017) emphasized that grit can efficiently stimulate efficacious education in English for Foreign or Second Language Learners (EFL/ESL) who are supposed to dedicate high levels of consideration to English. Although, grit has been examined in other realms, particularly in psychology (e.g., Duckworth et al., 2007, 2009), few studies have regarded it among EFL learners. Undeniably, few inquiries have investigated the rapport between grit and language acquisition, regardless of grit’s noticeable part in language learning in comparison to other issues (MacIntyre et al., 2019b). Despite the wide range of research undertaken to date, to the best of the researcher’s knowledge, the effect of grit on language engagement is not presupposed.

## Grit

Grit comprises two lower-order segments, the persistence of exertion and constancy of well-being (Duckworth et al., 2007). The primary sub-construct, persistence of exertion, shows a capacity to keep up with exertion for a long time notwithstanding challenges or disappointment. The second sub-concept, constancy of interests, alludes to the capacity to support comforts for a long period notwithstanding difficulties or misfortunes.

Grit is a moderately new hypothesis created by Duckworth (2016) that associates the constructs of zeal and determination to one’s capacity to effectively reach their objectives and Duckworth (2016) asserted that grit clarifies why some are successful in rushing into their objectives, while others are not. Grit is the energy one needs to stay with long-term life objectives regardless of the hardships, disappointments, or difficulties experienced (Duckworth and Gross, 2014; Duckworth, 2016). Grittier people see life as a long-distance race and show a solid hard-working attitude and responsibility. The construct of grit does not propose that people do not encounter disappointments or misfortunes, but that they can remain on track and press forward toward their definitive objective. Grit develops over a long period of figuring out how to manage and move past dismissal and disappointment (Duckworth, 2016). It is created as an individual learns the contrast between low-level objectives and higher-level objectives and figures out where to put their energies. Duckworth (2016) believed that it is not the ability that makes an individual gritty, rather the eagerness to continue learning and developing through one’s zeal for a movement. Duckworth (2016, p. 42) expressed, “talent is how quickly your skills improve when you invest effort.” When these improved abilities are used, accomplishment develops. The exclusive requirements set upon instructors highlight the pertinence of grit as a significant personality characteristic (Robertson-Kraft and Duckworth, 2014).

Educational psychologists (e.g., Duckworth et al., 2007; Strayhorn, 2014) have presented an intense attentiveness in grit and its inspiration on learners in scholastic situations and findings proved that gritty students commonly outperformed in exams than less gritty learners. Grittier learners also display developed educational hopes and show developed degrees of education from schools (Eskreis-Winkler et al., 2014). Although, Ivcevic and Brackett (2014) have not reported any relation between grit and learners’ accomplishment, the meta-analysis done by Credé et al. (2017) represented a positive correlation between students’ grittiness and their educational presentation.

## Implications and Future Directions

The current minireview tried to focus on the considerate aspect of grit in the EFL setting, particularly about learning engagement. In line with applied perception, it is suggested that EFL students may take advantage of activities or mediations intended to enhance constructive behaviors, such as grit and consistency. Within the EFL setting, some tasks and actions simplifying grit may be prepared to boost learners to set long-term goals constructed on their benefits. To accomplish these objectives, practice is required particularly concerning the mechanisms of failure and obstacles, teaching students how to hold failure as a prospect to be engaged.

The review provides fundamental proof about the significance of considering the settings in which learners are establishing their grit. It is overcritical that instructors, researchers, and authorities change from a reductionist perspective of students’ grit, with which the learners are seen as either gritty or not to reflect whether class settings care for all students’ grit. This is primarily precise for learners who face wide degrees of difficulties in their normal lives and so may have extreme grit. If grit should be engaged in instructive settings, instructors and policymakers need to discover significant methodologies of evaluating and reinforcing learners’ grit. Scholars, specifically, have a pledge to inspect grit regarding learner results as well as in terms of students’ more extensive conditions.

A noticeable distinctive of grit is its flexibility (Clark and Malecki, 2019), which means grit can be amended by involvement in the classes. In the SLA setting, the flexibility of grit can arrange for L2 teachers with an instrument to make their learners overcome the probable obstacles they might come upon throughout the L2 procedure. It could accordingly be maintained that regardless of the significant role of grit in learners’ educational success, it has not been satisfactorily premeditated, especially in the EFL setting. As a result, mastery of the role of grit deals with new standpoints for higher teaching supervisors when bearing in mind the students’ academic presentation. Grit is a disposition attribute that can be fostered and taught to scholars (Ebadi et al., 2018). Higher academic administrators can improve grit in students by providing chances to undertake long-term purposes worthy of learners’ determination and providing a demanding and compassionate setting to accomplish their objectives. Instructors and experts could accentuate fulfillment with peer relationships in the development of instruction by encouraging supportive and collaborative peer tasks and actions that enable group cooperative learning to support them in the process of learning, surge their degree of grit and increase their educational presentation, engagement, and determination (Lan and Moscardino, 2019; Derakhshan, 2021; Xie and Derakhshan, 2021).

Grit has got significant consideration as an important personality aspect that needs to be reinforced in youth and has been acknowledged divergent from care in its multifaceted confidence on both attention and strengths (Duckworth et al., 2007). As grit involvements and correlated experimental investigations are still emergent, more studies are compulsory regarding if grit is to some degree teachable in school or not, and if so, focus on the suggested approach to teach it. These are the current issues that must be taken into account by researchers and scholars.

## Author Contributions

The author confirms being the sole contributor of this work and has approved it for publication.

## Conflict of Interest

The author declares that the research was conducted in the absence of any commercial or financial relationships that could be construed as a potential conflict of interest.

## Publisher’s Note

All claims expressed in this article are solely those of the authors and do not necessarily represent those of their affiliated organizations, or those of the publisher, the editors and the reviewers. Any product that may be evaluated in this article, or claim that may be made by its manufacturer, is not guaranteed or endorsed by the publisher.
